# Effects of Bosentan on Hypoxia, Inflammation and Oxidative Stress in Experimental Blunt Thoracic Trauma Model

**DOI:** 10.3390/medicina60071148

**Published:** 2024-07-17

**Authors:** Nedim Uzun, Sinem Durmus, Gonca Gercel, Burhan Aksu, Naile Fevziye Misirlioglu, Hafize Uzun

**Affiliations:** 1 Department of Emergency, Gaziosmanpaşa Training and Research Hospital, University of Health Sciences, Istanbul 34098, Turkey; nedim.uzun@sbu.edu.tr; 2 Department of Medical Biochemistry, Faculty of Medicine, Katip Celebi University, Izmir 35620, Turkey; durmus.sinem@gmail.com; 3 Department of Pediatric Surgery, Istanbul Medeniyet University Göztepe Training and Research Hospital, Istanbul 34730, Turkey; goncagercel@gmail.com (G.G.); burhanfeyza@yahoo.com (B.A.); 4 Department of Biochemistry, Gaziosmanpaşa Training and Research Hospital, University of Health Sciences, Istanbul 34098, Turkey; nailemisirlioglu@gmail.com; 5 Department of Medical Biochemistry, Faculty of Medicine, Istanbul Atlas University, Istanbul 34408, Turkey

**Keywords:** pulmonary contusion, endothelin-1, ET-1, hypoxia-inducible factor-1 HIF-1, nuclear factor-kappa B, tumor necrosis factor-α pro-oxidant antioxidant balance, lung tissue

## Abstract

*Background and Objectives:* In this study, we aimed to investigate the effects of bosentan, an endothelin receptor antagonist, on endothelin-1 (ET-1), hypoxia-inducible factor-1 (HIF-1), nuclear factor-kappa B (NF-κB), and tumor necrosis factor (TNF)-α as inflammation markers, pro-oxidant antioxidant balance (PAB), and total antioxidant capacity (TAC) levels as oxidative stress parameters in lung tissues of rats in an experimental model of pulmonary contusion (PC) induced by blunt thoracic trauma. *Materials and Methods:* Thirty-seven male Sprague-Dawley rats were divided into five groups. C: The control group (*n* = 6) consisted of unprocessed and untreated rats. PC3 (*n* = 8) underwent 3 days of PC. PC-B3 (*n* = 8) received 100 mg/kg bosentan and was given orally once a day for 3 days. The PC7 group (*n* = 7) underwent 7 days of PC, and PC-B7 (*n* = 8) received 100 mg/kg bosentan and was given orally once a day for 7 days. *Results:* ET-1, NF-κB, TNF-α, HIF-1α, and PAB levels were higher, while TAC activity was lower in all groups compared with the control (*p* < 0.05). There was no significant difference in ET-1 and TNF-α levels between the PC-B3 and PC-B7 groups and the control group (*p* < 0.05), while NF-κB, HIF-1α, and PAB levels were still higher in both the PC-B3 and PC-B7 groups than in the control group. Bosentan decreased ET-1, NF-κB, TNF-α, HIF-1α, and PAB and increased TAC levels in comparison to the nontreated groups (*p* < 0.05). *Conclusions:* Bosentan decreased the severity of oxidative stress in the lungs and reduced the inflammatory reaction in rats with PC induced by blunt thoracic trauma. This suggests that bosentan may have protective effects on lung injury mechanisms by reducing hypoxia, inflammation, and oxidative stress. If supported by similar studies, bosentan can be used in both pulmonary and emergency clinics to reduce ischemic complications, inflammation, and oxidative stress in some diseases that may be accompanied by ischemia.

## 1. Introduction

Trauma is one of the leading causes of death, especially in the young population. There is a wide spectrum of thoracic traumas, ranging from simple isolated rib fractures to life-threatening injuries. The extent of the damage is directly proportional to the disruption of cardiac and pulmonary physiology [1]. Pulmonary contusion (PC), a multifaceted clinical entity, is characterized by hemorrhage into the interstitial space and alveoli within the lung and accompanying tissue reactions, such as leukocyte infiltration and edema. Although it usually occurs with blunt thoracic trauma, it can also occur with specific penetrating injuries, such as gunshot wounds. PC is an important cause of mortality and morbidity in patients with blunt chest trauma and multiple body trauma [2].

In PC that develops after blunt chest trauma, there is an increase in proinflammatory cytokines (such as IL-1β, IL-6, tumor necrosis factor (TNF)-α, and NO) and oxidative stress agents after the innate immune response. PC pathophysiology, including inflammation, is a risk factor for the development of acute lung injury and acute respiratory distress. Acute lung injury in human and animal models has been shown to be characterized by an intense inflammatory response in the lung parenchyma. The natural inflammatory response triggered by direct or indirect trauma to the lungs includes leukocyte activation in the blood, macrophage activation in the tissue, production of different mediator series, including cytokines, chemokines, oxygen radicals, arachidonic acid metabolites, and the complement and coagulation cascade [3].

Hypoxia-inducible factor-1 (HIF-1) plays a vital role in the metabolic reprogramming of cells in a hypoxic environment. HIF-1 is involved in the chronic inflammatory response by showing an increase secondary to nuclear factor-kappa B (NF-κB). As a result, the stimulatory or suppressive effects of HIF-1 on inflammation vary according to time and space. Therefore, it assumes different roles at various stages of inflammation. HIF-1α expression in alveolar epithelial cells increases lung inflammation in an NF-kB-mediated manner and promotes cell-mediated inflammation (CD4+ CD8+) and proinflammatory cytokines that proportionally downregulate CD55 and increase complement-mediated endothelial damage [4,5].

Maintaining the prooxidant-antioxidant balance (PAB) in an organism is a multifactorial event involving many metabolic pathways in the cell. Increases in free radical formation affect macromolecules such as lipids, proteins, and DNA in the organism and lead to changes in the structure and functions related to them and, consequently, cell damage [6]. The organism has a powerful defense system that prevents oxidative stress damage. The inflammatory response is one of the most important factors determining prognosis after PC. Oxidative stress, antioxidant defense, and inflammatory cytokine levels, which are indirect prognostic factors related to lung trauma, have been investigated [7,8,9].

Endothelin-1 (ET-1) binds to endothelin receptors (A and B) in the pulmonary vasculature and causes vasoconstriction. Bosentan, ambrisentan, and masitentan are endothelin receptor antagonists used in the treatment of pulmonary arterial hypertension (PAH) [10]. The efficacy of bosentan in the BREATHE-1 study has been shown [11]. In this study, bosentan prevented the worsening of the 6 min walk distance (6 MWD) distance, although not as much as in idiopathic (IPAH) patients.

Since bosentan, which is an inhibitor of both ET-A and ET-B receptors, may play an important role in inflammatory diseases and act as a regulator of various steps of inflammatory and oxidation/reduction reactions, it was thought that it might be effective in preventing or reversing the inflammatory reactions that develop after lung contusion caused by blunt thoracic trauma. In this study, we investigated the effects of bosentan on ET-1, HIF-1, NF-κB, and TNF-α as inflammation markers and PAB and total antioxidant capacity (TAC) as oxidative stress parameters in the lung tissues of rats in an experimental model of PC induced by blunt thoracic trauma. 

## 2. Materials and Methods

All experiments were approved by the Yeditepe University (2017/601) Ethics Committee for Animal Experiments and followed the NIH Guide for the Care and Use of Laboratory Animals. This work is derived from Gonca Gercel’s Specialist’s Thesis (Investigation of bosentan’s effects on pulmonary contusion created by blunt thoracic trauma in rats) [12].

### 2.1. Animals 

Thirty-seven Sprague-Dawley rats, weighing from 200 to 250 g, were included in this study under standard laboratory conditions (22 ± 1 °C, 12-h light/dark cycle). They were fed with standard rat chow and tap water ad libitum.

### 2.2. Experimental Protocol

**Figure 1:**
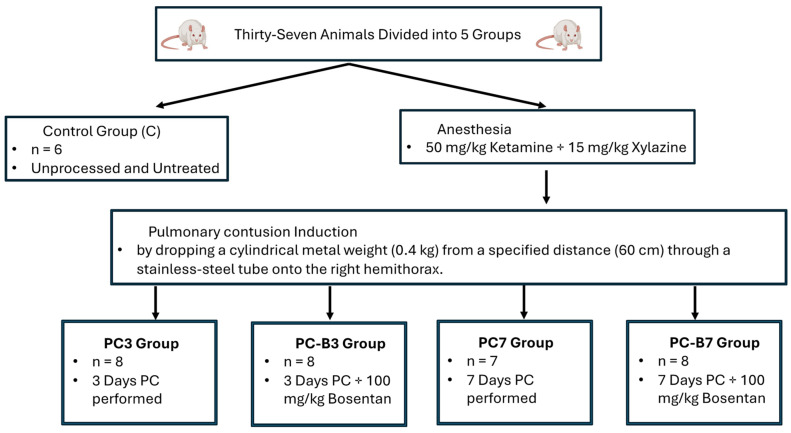
Flowchart of the experimental protocol.

Thirty-seven animals were randomly divided into 5 groups, and all 4 groups, except for the control group, underwent PC. In the first group, C, the control group (*n* = 6) consisted of unprocessed and untreated rats. The second group, PC3 (*n* = 8), underwent PC for 3 days. The third group, PC-B3 (*n* = 8), received 100 mg/kg bosentan and was given orally once a day for 3 days. The fourth group, the PC7 group (*n* = 7), underwent 7 days of PC, and the fifth group, PC-B7 (*n* = 8), received 100 mg/kg bosentan and was given orally once a day for 7 days (Figure 1). 

All rats were anesthetized with intraperitoneal ketamine (50 mg/kg) and xylazine (15 mg/kg). PC was induced by dropping a cylindrical metal weight (0.4 kg) from a specified distance (60 cm) through a stainless steel tube onto the right hemithorax. In this way, the trauma was standardized by applying 2.35 J energy on the chest according to the formula E = mgh (E = energy [joule], m = mass of the cylinder [kg], g = gravity constant [9.8 m/s^2^], and h = height [meter]). This is a modified form of the model proposed by Raghavendran et al. [13].

### 2.3. Biochemical Analysis Lung Tissue

Lung tissue samples were collected for subsequent biochemical analysis, snap-frozen in liquid nitrogen, and then stored at −80 °C. Lung tissues were homogenized using 20% phosphate buffer via a homogenizer (Next Advance Bullet Blender Storm 24). The homogenates were centrifuged for 10 min at 3.000× *g* and 40 °C to remove debris. The supernatant was collected for the determination of ET-1, NF-κB, TNF-α, HIF-1α, TAC, and PAB. All samples were examined twice.

The levels of ET-1, NF-κB, TNF-α, and HIF-1α were measured using commercially available ELISA (MyBioSource, Inc. in San Diego, CA, USA), and the manufacturer’s instructions were followed for the assessment. 

### 2.4. Measurement of Lung Tissue Endothelin-1 (ET-1)

Lung tissue ET-1 levels were measured using commercially available ELISA (MyBioSource, Inc. in San Diego, CA, USA), and the manufacturer’s instructions were followed for the assessment. The coefficients of intra- and inter-assay variation were 7.0% (*n* = 20) and 8.4% (*n* = 20), respectively.

### 2.5. Measurement of Lung Tissue Nuclear Factor-Kappa B (NF-κB)

Lung tissue NF-κB levels were measured using commercially available ELISA (MyBioSource, Inc. in San Diego, CA, USA), and the manufacturer’s instructions were followed for the assessment. The coefficients of intra- and inter-assay variation were 7.1% (*n* = 20) and 8.2% (*n* = 20), respectively.

### 2.6. Measurement of Lung Tissue Tumor Necrosis Factor (TNF)-α 

Lung tissue TNF-α levels were measured using commercially available ELISA (MyBioSource, Inc. in San Diego, CA, USA), and the manufacturer’s instructions were followed for the assessment. The coefficients of intra- and inter-assay variation were 7.3% (*n* = 20) and 8.5% (*n* = 20), respectively.

### 2.7. Measurement of Lung Tissue Hypoxia-Inducible Factor-1 (HIF-1)-α

Lung tissue HIF-1α levels were measured using commercially available ELISA kits (MyBioSource, Inc., San Diego, CA, USA), and the manufacturer’s instructions were followed for the assessment. The coefficients of intra- and inter-assay variation were 8.1% (*n* = 20) and 9.6% (*n* = 20), respectively.

### 2.8. Measurement of Lung Tissue Prooxidant-Antioxidant Balance (PAB)

Lung tissue PAB was measured with the method of Alamdari et al., [14] with slight modifications. The oxidation-reduction indicators used in this method were 3,3′,5,5′-tetramethylbenzidine (TMB), and TMB cations, which have different optical and electrochemical properties. The coefficients of intra- and inter-assay variation were 5.0% (*n* = 20) and 6.2% (*n* = 20), respectively.

### 2.9. Measurement of Ferric Reducing Antioxidant Power (FRAP)

The antioxidant status of the serum samples was measured with the FRAP assay, which is a redox-linked colorimetric method that uses reductant antioxidants [15]. The coefficients of intra- and inter-assay variation were 4.9% (*n* = 20) and 6.1% (*n* = 20), respectively. 

### 2.10. Statistical Analysis

Statistical analysis was conducted using JASP 0.18.3. Descriptive statistical methods, including calculation of the mean and standard deviation, were employed in the evaluation of the study data. The analysis of variance (ANOVA) test was used for the comparison of groups, with the Tukey test serving as a post-hoc test. The threshold for statistical significance was set at *p* < 0.05.

## 3. Results

**Figure 2:**
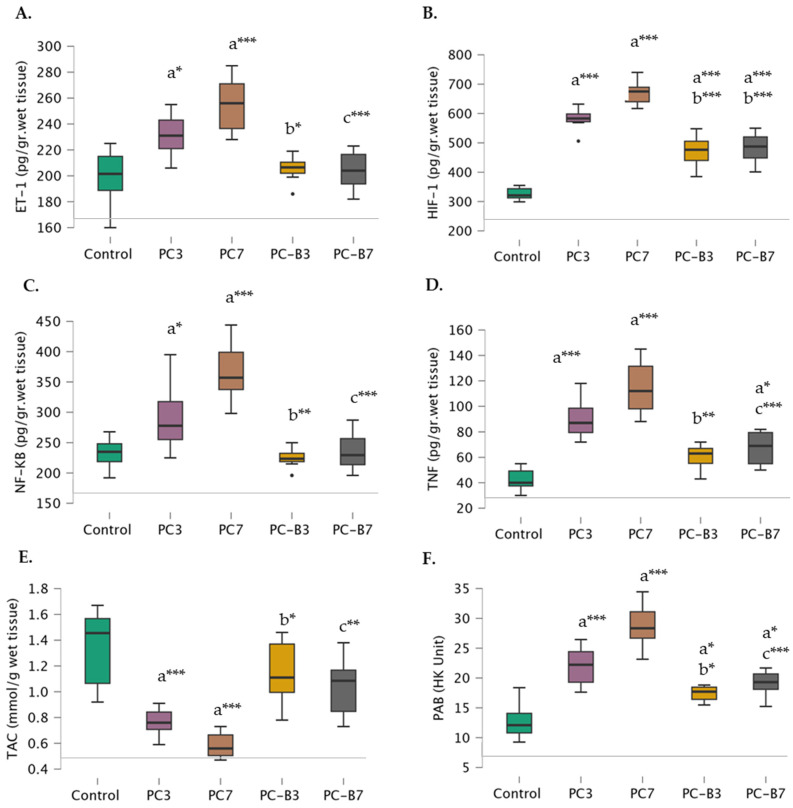
Comparative Levels of Rat Lung Tissue (**A**) Endothelin-1 (ET-1), (**B**) Hypoxia-Inducible Factor-1 (HIF-1)-α, (**C**) Nuclear Factor-kappa B (NF-κB), (**D**) Tumor Necrosis Factor (TNF)-α, (**E**) Ferric Reducing Antioxidant Power (FRAP) and (**F**) Prooxidant-Antioxidant Balance (PAB)across Control (*n* = 6), 3 Days Pulmonary contusion (PC) Performed (PC3, *n* = 8), PC3 with 100 mg/kg Bosentan (PC-B3, *n* = 8), 7 Days PC Performed (PC7, *n* = 7), and PC7 with 100 mg/kg Bosentan (PC-B7, *n* = 8) groups. Significant differences are indicated as follows: “a” indicates a significant difference compared to the control group, “b” indicates a significant difference compared to the PC3 group, and “c” indicates a significant difference compared to the PC7 group. * Means *p* < 0.05, ** means *p* < 0.01, and *** means *p* < 0.001.

As anticipated, ET-1 levels were found to be significantly higher in the PC-3 (231.38 ± 16.58) and PC-7 groups (254.86 ± 22.05) compared to the control group (198.67 ± 23.58). When comparing the bosentan-treated groups, it was observed that the PC-B3 group (205.50 ± 10.14) exhibited significantly lower ET-1 levels than the PC-3 group, whereas the PC-B7 group (204.00 ±14.46) displayed significantly lower ET-1 levels than the PC-7 group. Furthermore, there was no significant difference in ET-1 levels between the PC-B3 and PC-B7 groups and the control group (Figure 2A).

Additionally, the levels of HIF-1 were found to be lower in the control group (326.17 ± 22.15), PC-3 group (581.75 ± 37.44), and PC-7 group (670.14 ± 42.16). The level of this factor was found to be lower in the PC-B3 group (470.00 ± 56.10) than in the PC-3 group. Moreover, the level of HIF-1 was found to be lower in the PC-B7 group (670.14 ± 42.16) compared to the PC-7 group. However, its levels were still higher in both the PC-B3 and PC-B7 groups than in the control group (Figure 2B).

NF-κß levels were also elevated in the control (232.67 ± 26.91), PC-3 (293.63 ± 53.16), and PC-7 (367.43 ± 56.10) groups. However, a notable discrepancy was observed between the bosentan-treated groups. In fact, the levels in the PC-B3 group (224.75 ± 16.26) were found to be comparable to the control level and significantly lower than those observed in the PC-3 group. Similarly, in the PC-B7 group (235.00 ± 31.66), the levels were comparable to the control levels and significantly lower than those observed in the PC-7 group (Figure 2C).

TNF-α levels exhibited an increase in the PC-3 (90.25 ± 15.77), and PC-7 (114.86 ± 21.62) groups yet exhibited a notable decline in the PC-B3 group (60.50 ± 10.01) in comparison to the PC-3 group and a further decline in the PC-B7 group (67.25 ± 13.31) in comparison to the PC-7 group. Notably, while no significant difference was observed between the PC-B3 and controls, the PC-B7 group exhibited a higher level of TNF-α than the controls (Figure 2D).

TAC exhibited a gradual decline in the control (1.34 ± 0.32), PC-3 (0.79 ± 0.16), and PC-7 (0.59 ± 0.11) groups, while no significant difference was observed between the control and PC-B3 (1.15 ± 0.25) and PC-B7 (1.04 ± 0.23) groups. The PC-B3 group exhibited a significantly higher value than the PC-3 group, while the PC-B7 group demonstrated a significantly higher value than the PC-7 group (Figure 2E).

PAB levels exhibited a gradual increase in the control (12.82 ± 3.27), PC-3 (21.98 ± 3.24), and PC-7 (28.79 ± 3.82) groups, with levels being higher in the PC-B3 (17.44 ± 1.24) and PC-B7 (19.13 ± 2.14) groups compared to the control. However, they remained lower in the PC-B3 group compared to the PC-3 group and in the PC-B7 group compared to the PC-7 group (Figure 2F).

## 4. Discussion

Inflammation can be defined as tissue damage or a body’s unique response in the presence of inflammatory stimuli. In the current study, ET-1 levels were found to be significantly higher in the PC groups compared to the control group. The PC-B3 and PC-B7 groups exhibited significantly lower ET-1 levels than the PC-3 and PC-7 groups. Furthermore, there was no significant difference in ET-1 levels between the bosentan-treated groups and the control group. ET-1, one of the important endothelial synthesis products in systemic inflammation, plays a primary role in vascular tone regulation due to its tonic vasoconstrictor effect. ET-1-dependent constriction in response to vasodilator stimuli has a slow onset and persists for hours or even days. In addition to its vasoactive properties, it stimulates smooth muscle cell proliferation and contributes to vascular remodeling and leukocyte adhesion. Thus, it plays an important role in inflammation and atherogenesis [16]. Therefore, both the ET-A and ET-B receptor antagonists bosentan may provide therapeutic potential to reduce inflammatory responses in lung tissues in blunt thoracic trauma. Thus, although ET-1 was first identified in the vasculature, the effects of ET-1 go far beyond blood pressure control and appear to be essentially relevant to all major organs. While ET-1 serves many essential functions in the body, it is important to note that its dysregulation or overproduction can contribute to pathological conditions. Understanding the role of ET-1 is crucial for both basic science research and the development of therapeutic interventions in various diseases related to vascular function and inflammation. Researchers continue to study the complex regulatory mechanisms of ET-1 and its potential as a therapeutic target. While it is clear that many aspects of blunt thoracic trauma remain unexplained and ET-1 may play a role, this requires further investigation [16]. There is no study investigating ET-1 levels in lung tissues of rats in an experimental model of pulmonary contusion (PC) induced by blunt thoracic trauma. Researchers continue to study the complex regulatory mechanisms of ET-1 and its potential as a therapeutic target.

One of the most important factors determining the prognosis after lung contusion is the inflammatory response. Many changes occur in the lung parenchyma after contusion. These include hemorrhage, edema, and consolidation, which cause an impaired ventilation/perfusion ratio, hypoventilation, and decreased compliance, resulting in hypoxia [17]. The activation of HIF-1α plays a decisive role in cell proliferation and apoptosis. It is able to do this through its regulatory roles in ion changes in cells, transporters, circulating hormones, and their receptors [16,18]. In the current study, the levels of HIF-1 and NF-κB were found to be elevated in the control group, PC-3 group, and PC-7 group. The levels of HIF-1 and NF-κB were also found to be lower in the treated groups. However, their levels were still higher in the treated groups than in the control group. There is a well-known link between hypoxia and inflammation. Prominent among factors other than hypoxia that activate HIF-1α is NF-κB. NF-κB, in turn, can upregulate the transcription of HIFs [5,19]. In the current study, although no correlation was found between ET-1, NF-κB, and HIF-1α, transcriptional regulation of numerous hypoxia-responsive genes, including vascular endothelial growth factor (VEGF) and ET-1, is via the critical mediator of hypoxia-induced transcription, HIF-1α [20]. Panchal et al. [21] reported that new derivatives (17d, 16j, and 16h) of bosentan, an endothelin receptor antagonist, can dose-dependently reduce HIF-1α levels in vivo studies using monocrotaline (MCT) induced PAH in a rat model. PC often presents with hypoxemia, reduced lung compliance, and tachycardia. There are currently no specific pharmacological treatments [22]. Suresh et al. [22] PC results in profound global hypoxia with HIF-1α activation and subsequent upregulation of proinflammatory mediators, including IL-1β and IL-6. Our results indicate that blockade of HIF-1α activation with compounds represents a targeted therapy for blunt force trauma resulting in PC. Hypoxia is a direct consequence of PC and acid aspiration that prompts some patients to require mechanical ventilation [22]. 

TNF-α has been shown to play a key pathophysiological role in different models of acute lung injury [23,24]. Chu et al. [24] showed that TNF-α concentration increased in bronchoalveolar lavage (BAL) fluid and plasma in their acute lung injury models. In the current study, TNF-α levels were found to be significantly increased in contusion-induced rats. This result proves once again that TNF-α plays a role in contusion-induced lung injury. The results of the analysis showed that TNF-α levels measured 3 days after contusion increased further after 7 days. This suggests that TNF-α-mediated mechanisms are active in the early response after contusion, accompanied by a rapid progression of the inflammatory process. Bosentan had a significant effect on TNF-α, causing a decline. Bosentan suppresses TNF-α, a mediator that plays a key role in the inflammatory response induced by contusion, thus reflecting, at least through this mechanism, that it attenuates the destructive effects of contusion. Bellisai et al. [25] showed a decrease in profibrotic and proinflammatory cytokines levels in humans during treatment with bosentan. The degree of systemic inflammation and subsequent immunosuppression increases in relation to the rate of development of pulmonary organ failure [26].

It is known that alveolar macrophages can produce potent reactive oxygen and nitrogen species (ROS, RNS) called superoxide radicals and peroxynitrite in conditions such as PC, leading to acute lung injury [12,27]. These released ROS can cause oxidative damage through lipid peroxidation, protein oxidation, and DNA damage. In the current study, there was a significant increase in PAB and TAC levels in PC developing after blunt chest trauma. ROS and RNT contribute to inflammatory damage through both direct and indirect effects [28]. It was found that bosentan, a potent ET receptor antagonist, decreased oxidative stress by decreasing PAB levels and increasing TAC levels in the lung tissue. This result supports the positive effects of bosentan on the antioxidant system of PC.

### Limitation of Study

Other metabolic and hemodynamic markers, such as blood gas analysis and oxygen saturation monitoring, could not be evaluated in our study. 

## 5. Conclusions

In conclusion, PC has been shown to cause oxidative damage. ET-1 receptors may play an important role in PC-induced vasoconstriction, and this vasoconstriction could be reversed experimentally by inhibiting ET-1 receptors. Inhibition of ET-1 receptors with bosentan prevented contusion, and ET-1 levels decreased in the lungs. Bosentan, a potent ET receptor antagonist, has been found to reduce oxidative stress and inflammation in lung tissues. Bosentan was found to have anti-inflammatory and antioxidant effects in pulmonary contusion caused by blunt thoracic trauma. The low and high bosentan doses used in our study are within the recommended dose ranges according to the PC diagnosis and treatment guidelines, and studies with higher doses and long-term effects would be appropriate. Bosentan antagonizes the deleterious effects and hypoxic vasoconstriction of endothelin in contusion-induced lung injury and exhibits vasodilating antiproliferative, anti-inflammatory, and antioxidant effects. Accordingly, although bosentan shows multifactorial properties in PC, dose selection for clinical use in humans should be patient-based. If supported by similar studies, bosentan can be used in both pulmonary and emergency clinics to reduce ischemic complications, inflammation, and oxidative stress in some diseases that may be accompanied by ischemia.

## Data Availability

The data underlying this article are available in this article. If needed, please contact the corresponding author. The email address is huzun59@hotmail.com.
